# A modified HPLC method improves the simultaneous determination of plasma kynurenine and tryptophan concentrations in patients following maintenance hemodialysis

**DOI:** 10.3892/etm.2014.1512

**Published:** 2014-01-29

**Authors:** CHENGGEN XIAO, YUANHAN CHEN, XINLING LIANG, ZHEN XIE, MIN ZHANG, RUIZHAO LI, ZHILIAN LI, XIA FU, XIYONG YU, WEI SHI

**Affiliations:** 1 Nanfang Medical University, Guangzhou, Guandong 510080, P.R. China; 2 Division of Nephrology, Guangdong General Hospital, Guangdong Academy of Medical Sciences, Guangzhou, Guandong 510080, P.R. China; 3 Department of Dermatology, Sichuan Academy of Medical Sciences and Sichuan Provincial People’s Hospital, Chengdu, Sichuan 610000, P.R. China; 4 Department of Gastroenterology, The Sixth Affiliated Hospital, Sun Yat-Sen University, Guangzhou, Guangdong 510081, P.R. China; 5 Medical Research Center, Guangdong General Hospital, Guangdong Academy of Medical Sciences, Guangzhou, Guangdong 510081, P.R. China

**Keywords:** high-pressure liquid chromatography, kynurenine, tryptophan, hemodialysis, immunological biomarker, indoleamine 2, 3-dioxygenase

## Abstract

The ratio between plasma kynurenine (Kyn) and tryptophan (Trp) serves as a marker of indoleamine 2,3-dioxygenase, a critical immunomodulatory molecule. Simultaneous detection of the two markers may be performed using high-pressure liquid chromatography (HPLC). However, for uremic patients, the conventional detection method may be affected by a range of accumulated toxins. The current study aimed to establish a method for the simultaneous measurement of Kyn and Trp in patients following maintenance hemodialysis via HPLC-ultraviolet detection. The procedure involved the use of a SinoChrom ODS-BP C18 column (4.6×150 mm; inner diameter, 4.5 μm) and a mobile phase of 15 mmol/l sodium acetate acetic acid solution (containing 5% acetonitrile, pH 4.8). The modified method was verified using plasma samples from 10 healthy controls and 91 maintenance hemodialysis patients. The results demonstrated that the modified method was successful in simultaneously detecting the concentrations of Trp and Kyn in the healthy controls and maintenance hemodialysis patients. The method is simple, fast, accurate and suitable for clinical and research purposes in maintenance hemodialysis patients.

## Introduction

End-stage renal disease seriously affects human health and maintenance hemodialysis is the primary therapy for this condition. Although the technology for maintenance hemodialysis is continually being developed, malnutrition and inflammation significantly affect patient prognosis (1). Previous studies have shown that tryptophan (Trp) is an important regulator of immune homeostasis and inflammation (2). Trp is one of the essential amino acids in the human body and has a critical role in effective T cell function. Depletion of Trp activates effector T lymphocytes, thereby mediating immune tolerance. Furthermore, a metabolite of Trp, kynurenine (Kyn), promotes the generation of regulatory T lymphocytes, which further promote immune tolerance (3). Indoleamine 2,3-dioxygenase (IDO) is an intercellular rate-limiting enzyme that catalyzes the conversion of Trp to Kyn and plays a key role in immune homeostasis in uremic patients (2,4–6). IDO is also an immunoregulatory signal transduction molecule (7). Therefore, Kyn functions as a new potential marker in patients with end-stage renal disease.

**Figure 1:**
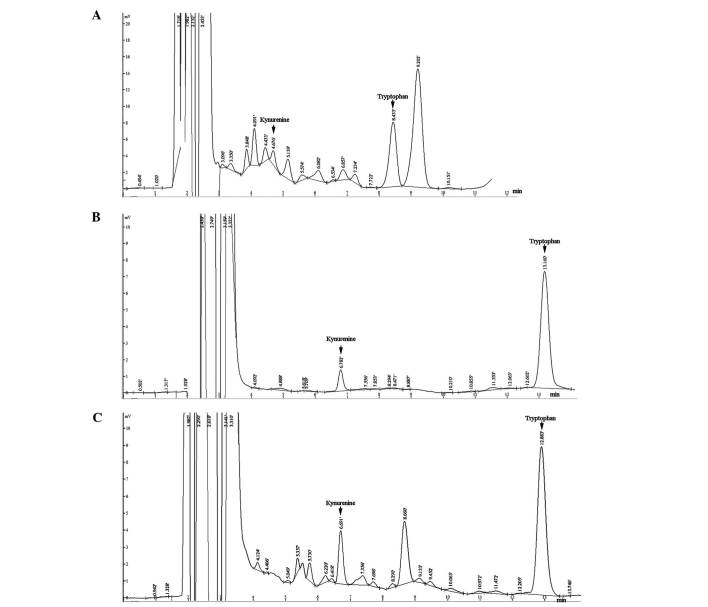
High-pressure chromatograms using the referenced and modified methods. Typical high-pressure chromatogram results using the (A) reference method for patients undergoing hemodialysis, (B) modified method for standard samples and (C) modified method for patients undergoing hemodialysis. Arrows indicate the peak positions of Trp and Kyn. Kyn, kynurenine; Trp, tryptophan.

IDO activity can be estimated by the concentration ratio of Kyn to Trp (5,8). The Kyn/Trp ratio correlates with inflammatory markers, including high-sensitivity C-reactive protein and soluble tumor necrosis factor-receptor-1, and has been recognized as a promising biomarker for evaluating immune homeostasis and inflammation (4,5). High-pressure liquid chromatography (HPLC) is a traditional method for measuring plasma amino acids levels. However, simultaneous measurement of Trp and Kyn is difficult using HPLC (9). Schefold *et al* reported a costly electrospray-tandem mass spectrometry assay for measuring Trp and Kyn simultaneously (5). With regard to clinical practice, several previous approaches to simultaneously measuring Trp and Kyn using HPLC have been reported (10,11). However, these methods were limited to isolating Kyn in uremic patients due to an interference of unknown uremic toxin (Fig. 1A). Additional HPLC methods have been used for uremic patients, but these methods have tedious and time-consuming procedures (12,13).

The present study aimed to develop a modified method to measure Trp and Kyn simultaneously in uremic patients for the evaluation of IDO activity.

## Materials and methods

### Reference method

The conventional method for simultaneous Trp and Kyn detection was used as the reference. A SymmetryShield RP-C18 column (150×3.9 mm; inner diameter, 5 μm) was purchased from Waters Corporation (Milford, MA, USA). The column had a mobile phase of 2.7% acetonitrile and pH 3.6 (11).

### Instruments

An LC-3A high-pressure liquid chromatograph (Shimadzu, Kyoto, Japan), SCI 100 UV-Vis detector (Daniel Elite Analytical Industries, Inc., Houston, TX, USA), YH-300 data processing system (Easeatech, Guangzhou, China) and VICILC 25-μl manual injector (Shimadzu, Kyoto, Japan) were used in the procedure.

### Reagents

Standard samples of Kyn and L-Trp were purchased from Sigma-Aldrich (St. Louis, MO, USA). Chromatographic pure acetonitrile, potassium dihydrogen phosphate, sodium acetate, analytical-grade acetic acid, 36% perchloric acid and ultra-pure water were treated with a Milli-Q Pure water device (Millipore Corporation, Billerica, MA, USA).

### Protein precipitant preparation

Perchloric acid solution at a volume fraction of 5.0% was prepared using ultra-pure water.

### Mobile phase

Acetic acid (36%) and acetonitrile solutions were used in the mobile phase. Sodium acetate crystals were weighed and sodium acetate-acetic acid solution (containing 5% acetonitrile) was prepared. Prior to use, the solution was ultrasonically degassed for 40 min, incubated overnight and filtered using a precolumn (KJO-4282; Phenomenex, Torrance, CA, USA).

### Preparation of the stock solution

Trp and Kyn stock solutions, at a concentration of 20 mmol/l, were prepared using 2.5% perchloric acid solution. Aliquotted stock solutions were then stored at −4°C. Standard working solutions were prepared using ultra-pure water prior to each use.

### Sample collection and processing

In total, 91 patients that were undergoing hemodialysis three times a week (single pool Kt/V, >1.2) were recruited from the Blood Purification Center at Guangdong General Hospital (Guangzhou, China). An additional 10 healthy volunteers were also recruited (Table I). Blood samples (2 ml) were collected from the arteriovenous fistulas of patients who had undergone maintenance hemodialysis and were placed into 4-ml EDTA tubes. After 1 h, the blood was centrifuged at room temperature at 2,200 × g for 10 min to separate the plasma. Blood sample collection was approved by the Ethics Committee of Guangdong General Hospital (2013069H) and all patients provided informed consent.

Plasma samples (50 μl) were placed into a centrifugal tube and the same volume of 5% perchloric acid solution was added. Next, the samples were placed in a vortex mixer for 30 sec and then maintained at room temperature for 15 min to allow plasma protein precipitation (11). Finally, the samples were centrifuged at 12,000 × g for 5 min and 20-μl samples of supernatant were collected for analysis.

### Chromatographic requirements

The procedure involved a SinoChrom ODS-BP C18 column (4.6×150 mm; inner diameter, 4.5 μm; Dalian Elite Analytical Instruments Co., Ltd., Dalian, China) and a mobile phase of 15 mmol/l sodium acetate acetic acid solution (containing 5% acetonitrile, pH 4.8) at a flow rate of 1.0 ml/min. The SC100-UV-visible detector was operated at 225 nm and the injection volume was 20 μl. Concentrations were measured at room temperature.

### Preparation of mixed plasma

In total, 20 plasma samples were selected randomly from the 91 patients undergoing maintenance hemodialysis. The samples were mixed well and stored in a −20°C freezer for later precision and recovery measurements (10).

### Qualitative and quantitative analyses for Kyn and Trp

Qualitative analyses for Kyn and Trp were performed using the peak retention value comparison and superposition methods, while quantitative analysis was performed using the external standard method. Data processing was conducted using a YH-300 Chromatograph workstation (Easeatech). Quantitative results are expressed as mean ± SD.

## Results

### Trp and Kyn concentrations

Concentrations of Trp and Kyn were detectable in all 10 healthy volunteers and in 90 of the 91 patients who underwent maintenance hemodialysis under the modified conditions (Table I). By contrast, the reference method only detected the concentrations of Trp and Kyn in 14 of the 91 patients undergoing maintenance hemodialysis. Failure of the reference method primarily arose from peak interference (Fig. 1A).

### Standard curve and equation

Standard working solutions were prepared by combining 2.5% perchloric acid solution with Trp and Kyn (Kyn, 0.004–100 μmol/l; Trp, 0.01–1,000 μmol/l). Each sample was injected three times and the average result was used for analysis. The results are shown in Table II, where y represents the peak area (μV.sec) and x is the sample concentration (μmol/l). A marked linear correlation was observed between the peak area and the sample concentrations of Kyn and Trp, with a wide linear range and a low detection limit, indicating good sensitivity.

### Precision test

Chromatographic columns and precolumns were washed daily with formaldehyde and the standard curves were corrected every two days (10). Intra- and inter-day precision tests were performed on mixed plasma samples (Table III).

## Discussion

Kyn/Trp ratio is a marker that reflects immune homeostasis and inflammation. Although existing HPLC methods are able to rapidly and simultaneously detect Trp and Kyn in non-uremic patients, they fail to do so for uremic patients due to the presence of unknown uremic substances that have absorption profiles similar to that of Kyn (Fig. 1). In the present study, the column diameter, pH value of the mobile phase and the content of acetonitrile were adjusted to isolate Trp and Kyn from these interfering components (Fig. 1C). The results indicated that the modified method was able to simultaneously detect the concentrations of plasma Trp and Kyn in uremic patients.

The method used in the present study differs from previously reported HPLC methods. Zhen *et al* and Koening *et al* used a 5-μm C18 column and adjusted the wavelength of the ultraviolet detector during detection (12,13). In the current study, a 4.5-μm C18 column was used. Furthermore, the composition of the mobile phase was adjusted, which allowed for the detection of Kyn and Trp simultaneously at a fixed wavelength and with a shortened detection time. In addition, the current method has a wider detection range which completely covers the levels of human plasma Trp and Kyn under normal and pathological states. The modified method is also applicable for *in vitro* detection. However, in the present study, Trp and Kyn levels remained undetected in one sample. The reason for this detection failure remains to be explored.

In conclusion, the modified HPLC method is able to detect Trp and Kyn simultaneously in uremic patients, satisfying the requirements for IDO determination in clinical practice.

## Figures and Tables

**Table I:** Characteristics of the control individuals and patients undergoing hemodialysis.

Characteristics	Controls	Patients
Age, years	34±10	53–77
Gender, male/female, n	5/5	51/40
Plasma Kyn concentration, μmol/l	0.8–0.4	1.07±0.10
Plasma Trp concentration, μmol/l	31.3±21.2	5.62±0.98
Plasma albumin, g/l		31.1±3.5
Hemodialysis time, days		1326.9±317.2
Single pool Kt/V*		1.5±0.3

Kyn, kynurenine; Trp, tryptophan.

**Table II:** Regression analysis and detection limits for Kyn and Trp.

Substance	Regression equation	R^2^	Linear range, μmol/l	Detection limit
Kyn	y = 28007x + 3843.9	1.0	0.08–50	0.02
Trp	y = 27331x + 85588	1.0	0.8–500	0.2

x, concentration (μmol/l). y, peak area (μV.sec). Kyn, kynurenine; Trp, tryptophan.

**Table III:** Precision of the intra- and inter-day measurements of Kyn and Trp (n=20).

Substance	Intra-day, mol/l	RSD, %	Inter-day, mol/l	RSD, %
Kyn	0.7301±0.0910	2.60	0.7415±0.0250	3.38
Trp	3.0280±0.0403	1.33	2.9184±0.1279	2.38

Values are expressed as mean ± SD. Kyn, kynurenine; Trp, tryptophan; RSD, relative standard deviation.
